# Detection and quantification of ezetimibe and its major glucuronide in patients with hepatic impairment via liquid chromatography-tandem mass spectrometry

**DOI:** 10.1016/j.jpba.2026.117455

**Published:** 2026-03-09

**Authors:** Dominique O. Farrera, Max M. Maloney, Christopher S. LaMadrid, Paige Lenzen-Hammerel, Lauren R. Radtke, Nathan J. Cherrington

**Affiliations:** Department of Pharmacology and Toxicology, College of Pharmacy, University of Arizona, Tucson, AZ 85721, USA

**Keywords:** LC–MS/MS, Ezetimibe (EZE), Ezetimibe glucuronide (EZEG), Biomarker, Protein precipitation, Hepatic impairment

## Abstract

Following administration of ezetimibe (EZE), levels of the glucuronidated metabolite ezetimibe-glucuronide (EZEG) are altered in patients with hepatic impairment. A rapid and sensitive liquid chromatography–tandem mass spectrometry method (LC-MS/MS) method was developed and validated to quantify EZE and EZEG in human plasma and urine. Samples were prepared using a protein precipitation procedure with methanol containing stable isotope-labeled internal standards (EZE-d_4_ and EZEG-d_4_). Separation of the analytes was achieved using acetonitrile–water (0.1% formic acid) as the mobile phase at a flow rate of 0.5 mL/min on a C18 column. The analytes were detected using negative ionization in multiple reaction monitoring (MRM) mode. The mass transition pairs of *m/z* 408.4→271.0 and *m/z* 584.5→271.0 were used to quantify EZE and EZEG, respectively. The method was linear over a concentration range of 1.5 ng/mL – 1 μg/mL for EZE and EZEG in the plasma. In the urine, EZE was detectable a linear range of 5 ng/mL – 1 μg/mL and EZEG ranged from 3 ng/mL – 1 μg/mL. The method was validated in accordance with U.S. Food and Drug Administration and European Medicines Agency regulatory standards, including specificity, sensitivity, stability, repeatability and reproducibility. This ensures accuracy and reliability of test results which thereby enhances patient safety and helps support clinical decisions.

## 1. Introduction

Hepatic impairment is a major clinical concern as it significantly impacts drug distribution, metabolism, and clearance, ultimately compromising both safety and therapeutic efficacy [1]. Regulatory agencies, such as the U.S. Food and Drug Administration (FDA) and the European Medicines Agency (EMA), recommend formal evaluation of pharmacokinetics in patients with hepatic impairment when hepatic metabolism or biliary excretion represents a major route of drug clearance [2]. While liver chemistry panels and clinical scoring systems such as the Child–Pugh classification remain standard for stratifying hepatic impairment, these approaches lack precision in determining drug-handling capacity [3,4]. As such, there is a need for exogenous probes that can directly and quantitatively reflect hepatic function.

Ezetimibe (EZE), an FDA approved drug, normally prescribed for the treatment of hyperlipidemia, represents one such candidate. Following oral administration, EZE undergoes extensive glucuronidation in both the liver and intestine, forming ezetimibe-glucuronide (EZEG) as its primary circulating metabolite [5]. In healthy individuals, EZEG is predominantly cleared via biliary excretion [5]. However, in patients with hepatic impairment, diminished hepatobiliary transport and reduced clearance result in altered systemic disposition of EZEG, leading to both elevated plasma concentrations and increased urinary recovery [6–9].

These pharmacokinetic shifts suggest that the disposition of EZE and EZEG may serve as a mechanistically informed and potentially regulatory-acceptable exogenous biomarker of hepatic function. Numerous methods exist for detection of EZE and EZEG in human plasma [10–14]. Despite this, no fully validated liquid chromatography–tandem mass spectrometry (LC–MS/MS) methods currently exist for the quantification of EZEG in human urine, a matrix that may be particularly informative in hepatic impairment studies. Furthermore, while one-step protein precipitation protocols have been explored for bioanalytical workflows, existing methods often rely on multistep extraction procedures or lack the sensitivity and robustness required for accurate measurement of polar glucuronide metabolites such as EZEG [15]. To address these gaps, we developed and validated a rapid, sensitive, and specific LC–MS/MS assay for simultaneous quantification of EZE and EZEG in both plasma and urine. Using an optimized one-step protein precipitation approach, this method achieves low limits of quantification, robust recovery, and excellent stability while meeting FDA and EMA regulatory acceptance criteria. This assay therefore provides a novel and reliable analytical platform to support pharmacokinetic evaluations of EZE in patients with hepatic impairment and to enable the exploration of EZEG as a clinically useful exogenous biomarker of hepatic function.

## 2. Materials and methods

### 2.1. Chemicals

All chemicals were purchased from Sigma-Aldrich (St. Louis, MO, USA) unless otherwise stated. EZE and ezetimibe-d4 (EZE-d4) were purchased from Cayman Chemicals (Ann Arbor, MI, USA). EZEG was purchased from Sussex Research (Ottawa, Canada). Ezetimibe-D-glucuronide-d4 (EZEG-d4) was purchased from Biosynth International (Louisville, KY, USA). HPLC-grade methanol, acetonitrile (ACN), formic acid, and water were obtained from Thermo Fisher Scientific (Pittsburgh, PA, USA).

### 2.2. Solution preparations

#### 2.2.1. Preparation of stock solutions and standards

Stock solutions of both EZE and EZEG were prepared at 1 mg/mL in 80% dimethyl sulfoxide (DMSO) from which the appropriate volumes were diluted with methanol to obtain different working solutions. Calibration standards and quality control (QC) samples were prepared by spiking blank plasma or urine with the appropriate working solutions at known concentrations. Stable isotope-labeled internal standards (IS), EZE-d4 and EZEG-d4, were prepared at 100 μg/mL and 1 μg/mL, respectively, in Methanol. Working standard solutions of the IS were prepared in Methanol at final concentrations of 25 ng/mL EZE-d4 and 10 ng/mL EZEG-d4 for plasma, and 50 ng/mL EZE-d4 and 10 ng/mL EZEG-d4 for urine) were prepared in methanol and used to precipitate protein. All stock and working solutions were stored at −20°C and were stable for 4 weeks except for the IS which were prepared fresh each week.

#### 2.2.2. Preparation of samples

A volume of 25 μL of plasma or 150 μL urine was pipetted into a microcentrifuge tube. Proteins were precipitated by adding a volume of 4:1 ice-cold Methanol containing the IS. Samples were vortexed and centrifuged at 14,000 × g for 15 min at 4 °C. The resulting supernatants were then transferred to vials and stored at −80 °C until analysis.

### 2.3. Instrumentation

#### 2.3.1. LC–MS/MS instrumentation and chromatographic conditions

Separation of the analytes was performed using an Agilent 1290 II UPLC system. Samples were maintained at 4°C in the autosampler and 4 μL were injected onto a Poroshell 120 EC-C18 column (50 × 3.0 mm, 2.7 μm particle size). The column temperature was maintained at 35 °C with a flow rate of 0.5 mL/min.

**Fig. 1.**
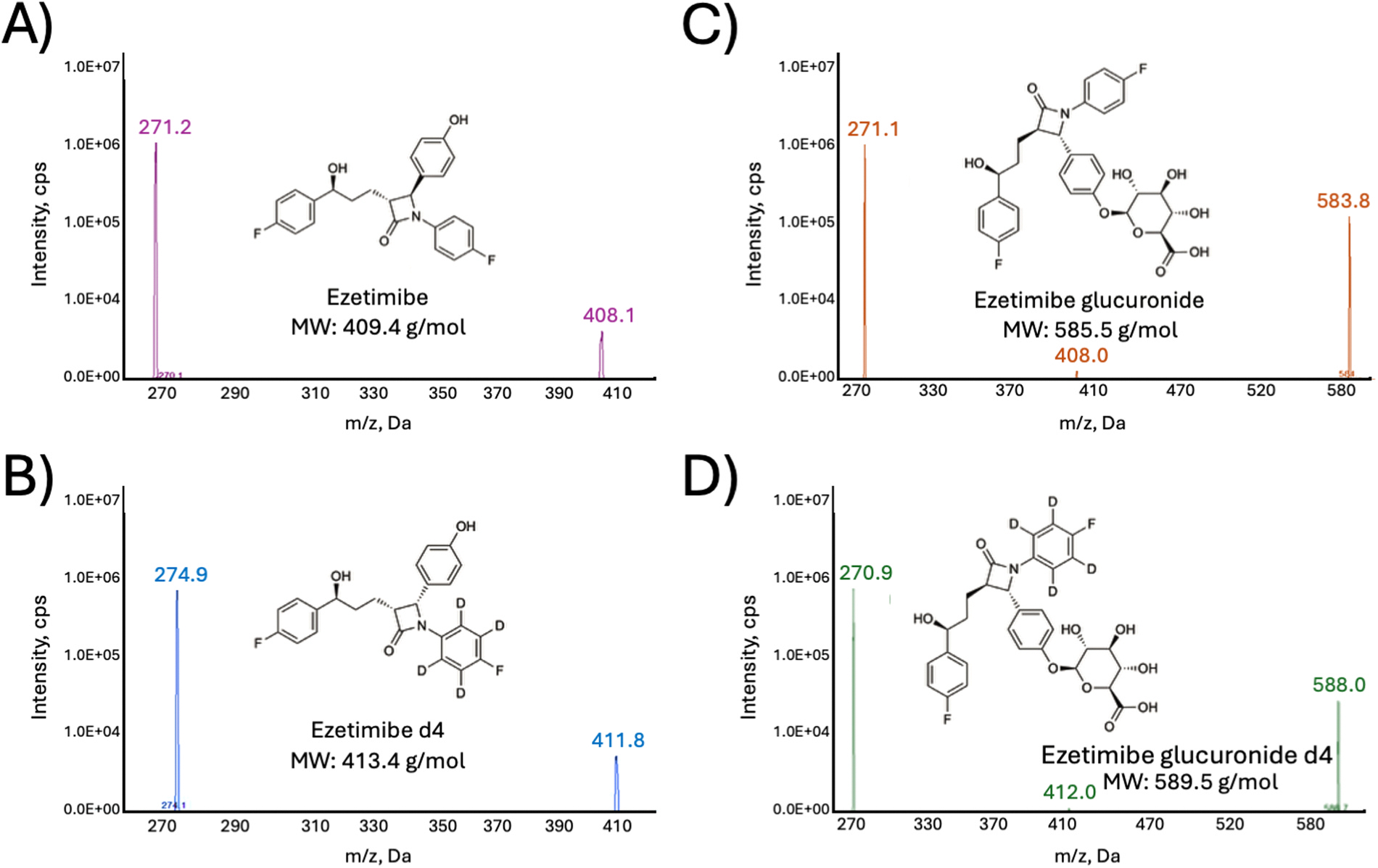
Product ion spectra of EZE, EZE d4, EZEG, EZEG d4. Spectra of deprotonated precursor ions were acquired in negative ESI mode on an AB SCIEX 6500 QTRAP mass spectrometer using the following source conditions: ion spray voltage, −5.5 kV; source temperature, 500 °C; nebulizer gas (N_2_), 20 psi; turbo gas, 40 psi; and collision gas, 9 psi. Pure solutions of each compound were directly infused at a concentration of 50 μg/mL. Panels show spectra and associated structures for (A) EZE, (B) EZE-d_4_, (C) EZEG, and (D) EZEG-d_4_, with structures displayed within the same panels as the spectra.

The mobile phase consisted of 0.1% formic acid in water (mobile phase A) and 0.1% formic acid in ACN (mobile phase B), with a gradient as follows: 5% B (0–0.5 min), 98% B (3–4.5 min), followed by a decrease to 5% B (5 min). The column was then held at 5% B (ACN + 0.1% formic acid) for 1.5 min The autosampler needle was washed prior to each injection using a 1:1 methanol:isopropanol solution. Scheduled multiple reaction monitoring (MRM) was performed in negative ion mode using an AB SCIEX 6500 QTRAP equipped with electrospray ionization (ESI) and the following source parameters: 5.5 kV ion spray voltage: 500 °C source temperature, 20 psi nebulizer gas (nitrogen), 40 psi turbo gas, and collision gas 9 psi. Pure solutions of each compound were directly injected to the mass spectrometer to obtain the appropriate MRM parameters. The declustering potential (DP) was optimized to achieve maximum signal intensity of the deprotonated molecular ion [M−H]^−^. Optimization was carried out using the ramp method available in SCIEX Analyst 1.6.3 software, which incrementally varied the DP while monitoring the ion signal response. The resulting profile was evaluated to determine the DP setting that produced the most stable and abundant precursor ion signal. Collision energy (CE) was similarly optimized by scanning a range of CE values during product ion scanning (Fig. 1). The fragmentation spectra were assessed to identify which CE provided the highest intensity for the selected product ions. These optimized transitions were then implemented in MRM acquisition methods to ensure high sensitivity and selectivity for each of the target compounds.

Consistent with WADA TD2023IDCR criteria, the following parameters were used for MS/MS analysis:

### 2.4. Method validation

The method was validated in accordance with FDA and EMA clinical diagnostic regulatory standards [11,16,17] including specificity, sensitivity, stability, repeatability and reproducibility to ensure accuracy and reliability of test results and help support clinical decisions.

#### 2.4.1. Specificity

Specificity of the method was assessed by analyzing chromatograms of blank plasma and urine from healthy volunteer donors for co-eluting peaks within the retention time windows for both EZE and EZEG to evaluate the potential interference from endogenous substances.

#### 2.4.2. Sensitivity and linearity

Linearity of the method in plasma was established using a calibration curve constructed by spiking blank plasma samples with EZE and EZEG at concentrations of 1.5, 5, 10, 50, 100, 500, and 1000 ng/mL for EZE and EZEG. Quality control (QC) samples at concentrations of 3 ng/mL (low concentration quality control, LQC), 500 ng/mL (medium concentration quality control, MQC), 900 ng/mL (high concentration quality control, HQC) for EZE and EZEG were prepared as described above. In the urine, calibration samples were prepared at 5, 10, 25, 50, 100, 250, 500, and 1000 ng/mL for EZE and 3, 15, 50, 100, 500, and 1000 for EZEG to establish linearity. Urine QC samples were prepared at concentrations of 15, 500, 850 for EZE and 12, 500, 850 for EZEG. To assess potential matrix effects, blank plasma and urine samples were used. Sensitivity of the method was determined using the lowest limit of quantification (LLOQ) which was reliably quantified with a signal-to-noise ratio over 10:1, accuracy between 80% and 120%, and precision (CV, %) under 20%. The calibration curves were then generated by plotting the ratio of the analyte peak area to ratio of the IS against the concentration of EZE or EZEG in plasma and urine. The resulting data were fitted using a linear regression model with a 1/x weighting factor in MultiQuant 3.0.2 software. All calibrators were prepared fresh for each run.

#### 2.4.3. Precision and accuracy

To determine the precision and accuracy of the method in plasma and urine, the respective QC samples (detailed above) along with LLOQ samples, were assessed in replicates of 5 (for within-run precision) on 4 different days to assess between-run precision. The accuracy of the method was determined by comparing the measured concentrations of EZE and EZEG in the QC samples of both urine and plasma to their known concentrations, multiplied by 100 to calculate the percentage of recovery. Accuracy between 80% and 120% was considered acceptable. Precision of the method for both plasma and urine were established using a coefficient of variation (CV, %) under 20%.

#### 2.4.4. Stability

Stability in plasma and urine was also evaluated using QC samples under various conditions. Short-term stability was assessed in both matrices after storage at room temperature for 24 h whereas long-term stability was evaluated by analyzing plasma and urine QC samples after storage at −80 °C for 3 months. Stability in both matrices after three freeze-thaw cycles was also assessed. Lastly, post-preparation stability was determined by analyzing samples after storage in the autosampler held at 4 °C for 24 h. Samples were considered stable if the measured concentrations were between 85% and 115% of freshly prepared standards. All stability experiments were repeated in triplicate with an n = 5.

### 2.5. Application of the method

The method was applied after participants received a single 1 mg oral EZE dose following an overnight fast and a ≥ 3-day washout from EZE-containing products. Blood was collected pre-dose and at 15, 30, 45, 60-, 120-, 180-, and 360-min post-dose into heparinized tubes, centrifuged within 30 min for separation of plasma, then stored at −80 °C for LC–MS/MS analysis. Urine was collected cumulatively over 24 h, total volume recorded, and 1 mL aliquots stored at −80 °C.

### 2.6. Ethical approval

All procedures involving human participants were conducted in accordance with the Declaration of Helsinki and were approved by the University of Arizona Institutional Review Board (protocol number: 1809927036). Written informed consent was obtained from all participants prior to study enrollment.

## 3. Results and discussion

### 3.1. LC–MS/MS method optimization

Due to the carboxyl group on EZEG, the method was initially optimized in negative ion mode. Positive ion mode was also assessed for EZE, however negative mode yielded higher peak intensities. The decision to prioritize simultaneous detection of EZE and its glucuronide metabolite was driven by the relevance of EZEG as a dominant circulating metabolite and its potential application as a biomarker reflecting hepatobiliary metabolic and transporter function. The mass transitions of *m/z* 408.4→271.0, *m/z* 584.5→271.0, 412.4→275.0 and *m/z* 588.5→271.0 were used to quantify EZE, EZEG, EZE-d4, and EZEG-d4, respectively (Table 1). Separation of the analytes was achieved using a mobile phase system of water–acetonitrile (ACN) + 0.1% formic acid. This mobile phase system provided optimal chromatographic performance, as the water-ACN allowed for effective separation of the analytes with sharp peak shapes. The addition of formic acid enhanced ionization efficiency. A system using methanol–water + 0.1% formic acid was also tested; however, this resulted in more background noise and a lower signal-to-noise ratio. A simple, segmented gradient was applied as follows; 5% B (0–0.5 min), 98% B (3–4.5 min), followed by a decrease to 5% B (5 min), then held at 5% B for 1.5 min. The high-organic hold was necessary to efficiently elute the highly lipophilic parent compound EZE while maintaining adequate retention of the more polar EZEG.

### 3.2. Optimization of extraction method and sample preparation

The analytical method was developed with the objective of enabling rapid and reproducible quantification of EZE and EZEG in human plasma and urine. A one-step protein precipitation protocol was therefore selected to balance analytical performance with throughput and operational simplicity, which are critical considerations for clinical studies. More complex extraction techniques, including liquid-liquid extraction and solid-phase extraction (SPE) were not assessed due to increased processing time, higher resource requirements, and reduced suitability for large-scale sample analysis. Protein precipitation with organic solvents to extract EZE and EZEG from human plasma and urine samples presents a streamlined clinical sample preparation approach. Its effectiveness for extraction of polar glucuronide metabolites however, has been debated due to potential matrix effects and reduced extraction efficiency. While Bahrami et al. [10] described a sensitive and specific method in which methanol protein precipitation was used to extract EZE from human serum, Guo et al. [12] reported serious matrix effects when using both methanol and ACN for protein precipitation of EZE and EZEG from human plasma. Further, Guo et al. [12] describe poor extraction recovery of highly polar and water-soluble compounds, such as glucuronide conjugates. Similarly, previously reported LC–MS/MS assays have quantified free and total EZE in human plasma using liquid–liquid extraction (LLE), demonstrating acceptable precision and sensitivity [18]. However, LLE approaches require more extensive sample handling and are less amenable to high-throughput analysis. Given the clinical relevance of EZEG, systematic optimization experiments were undertaken to ensure that protein precipitation could be applied without compromising analytical robustness.

Solvent composition (ACN, methanol, 80:20 methanol:water, and acetone), precipitation temperature, and incubation time were systematically evaluated. Initially, extraction with ACN, methanol, 80:20 methanol:water, and acetone were tested for both plasma and urine. We found that in the plasma, methanol yielded the highest analyte recovery for both EZE and EZEG. In the urine, however, ACN and methanol yielded comparable recoveries for EZEG, while methanol again produced the highest recovery for EZE. The use of 80:20 methanol:water was similarly effective as 100% methanol yet the sample-to-sample variability and within run variability was considerably higher. Lastly, acetone precipitation resulted in high background noise. We therefore opted for the use 100% methanol protein precipitation.

To optimize precipitation conditions, four temperature and incubation time conditions were evaluated: precipitation at room temperature without incubation, precipitation on ice without incubation, precipitation at −20 °C with overnight incubation, and precipitation at −80 °C with a 1 h incubation. In all cases, ice-cold methanol was added to samples, followed by vortex mixing and centrifugation at 14,000 × g for 15 min at 4 °C. The resulting supernatants were analyzed via LC-MS/MS to assess extraction efficiency and matrix cleanliness. Precipitation at −20°C overnight with overnight incubation produced the highest and most consistent recoveries for both analytes, indicating effective protein removal while minimizing the loss of polar compounds such as EZEG. Precipitation room temperature produced acceptable but lower recoveries, while −80°C for 1 h led to incomplete protein precipitation and reduced analyte recovery, indicating insufficient precipitation time.

To further preserve analyte stability, acidic conditions were maintained during sample processing to prevent cleavage of the glucuronide group from EZEG by use of water-ACN + 0.1% formic acid as the mobile phase. These optimizations collectively enable the method to achieve detection limits as low as 1.5 ng/mL in human plasma for both EZE and EZEG. In the urine, EZE and EZEG could be detected and quantified as low as 5 and 3 ng/mL, respectively, demonstrating that, despite its potential drawbacks, methanol-based protein precipitation can be a reliable and highly effective sample preparation technique for the extraction of EZE and EZEG.

### 3.3. Specificity and matrix effects

The method demonstrated high specificity for both EZE and EZEG in human plasma and urine. No co-eluting endogenous peaks were observed in the retention time windows of either analyte in blank plasma or urine samples, confirming the absence of interference. Matrix effects were formally evaluated during validation using blank plasma and urine obtained from multiple individual donors. No significant ion suppression or enhancement was detected at the retention times of EZE, EZEG, or their corresponding internal standards. The use of stable isotope-labeled IS (EZE-d4 and EZEG-d4) further compensated for potential matrix-related variability. Consistent recovery, stable retention times, and reproducible peak shapes across matrices confirmed that matrix effects were effectively controlled under the optimized conditions. Supplemental Figure 1 shows representative chromatograms of blank plasma, plasma spiked 5 ng/mL EZE and EZEG along with their respective IS, as well as plasma from volunteers with unknown levels of hepatic impairment following a 1 mg dose of oral EZE. Similarly, Supplemental Figure 2 shows representative chromatograms of blank urine, urine spiked 7.5 ng/mL EZE and EZEG along with their respective IS, as well as urine from the same group of volunteers.

### 3.4. Sensitivity and linearity

The method demonstrated high sensitivity and strong linearity for EZE and EZEG in human plasma and urine. In plasma, the method was linear over a concentration range of 1.5–1000 ng/mL for both EZE and EZEG. In urine, linearity was established from 5 to 1000 ng/mL for EZE and 3–1000 ng/mL for EZEG. Calibration curves were generated using linear regression with a 1/x weighting factor, and correlation coefficients (r^2^) of ≥ 0.99 or greater for all analytes.

### 3.5. Precision and accuracy

The accuracy and precision of the assay are summarized in Tables 2 and 3. In the plasma, the within-run accuracy was between 95–114% for EZE and 92–105% for EZEG, with within-run precision (CV) of 4–11% and 4–9%, respectively. Between-run accuracy in plasma ranged from 99 to 104% for EZE and 98–101% for EZEG, with between-run precision (CV) of 6–8% and 6–8%, respectively. Whereas, in the urine, within-run accuracy was ranged from 92 to 100% for EZE and 92–109% for EZEG and within-run precision (CV) of 3–15% and 6–13%, respectively. Between-run accuracy for EZE ranged from 97 to 100% and 98–105% for EZEG, with between-run precision (CV) of 8% and 10%, respectively. All values fell within acceptable limits for bioanalytical method validation.

### 3.6. Stability

Stability of EZE and EZEG was systematically evaluated in both plasma and urine LLOQ and QC samples across multiple storage and handling conditions, with stability defined as measured concentrations within 15% of nominal values. In plasma (Supplemental Table 1), both analytes were stable in the autosampler at 4 °C for at least 24 h, following three freeze–thaw cycles, and during short-term storage at room temperature for up to 12 h. Long-term stability in plasma was also confirmed for at least 90 days at −80 °C. In urine (Supplemental Table 2), EZE and EZEG demonstrated comparable stability under autosampler, freeze–thaw, short-term, and long-term storage conditions. All stability results met the regulatory acceptance criteria for bioanalytical method validation. Together, these results support the use of the method for overnight assays and reanalysis of stored samples

### 3.7. Application of the method

**Fig. 2.**
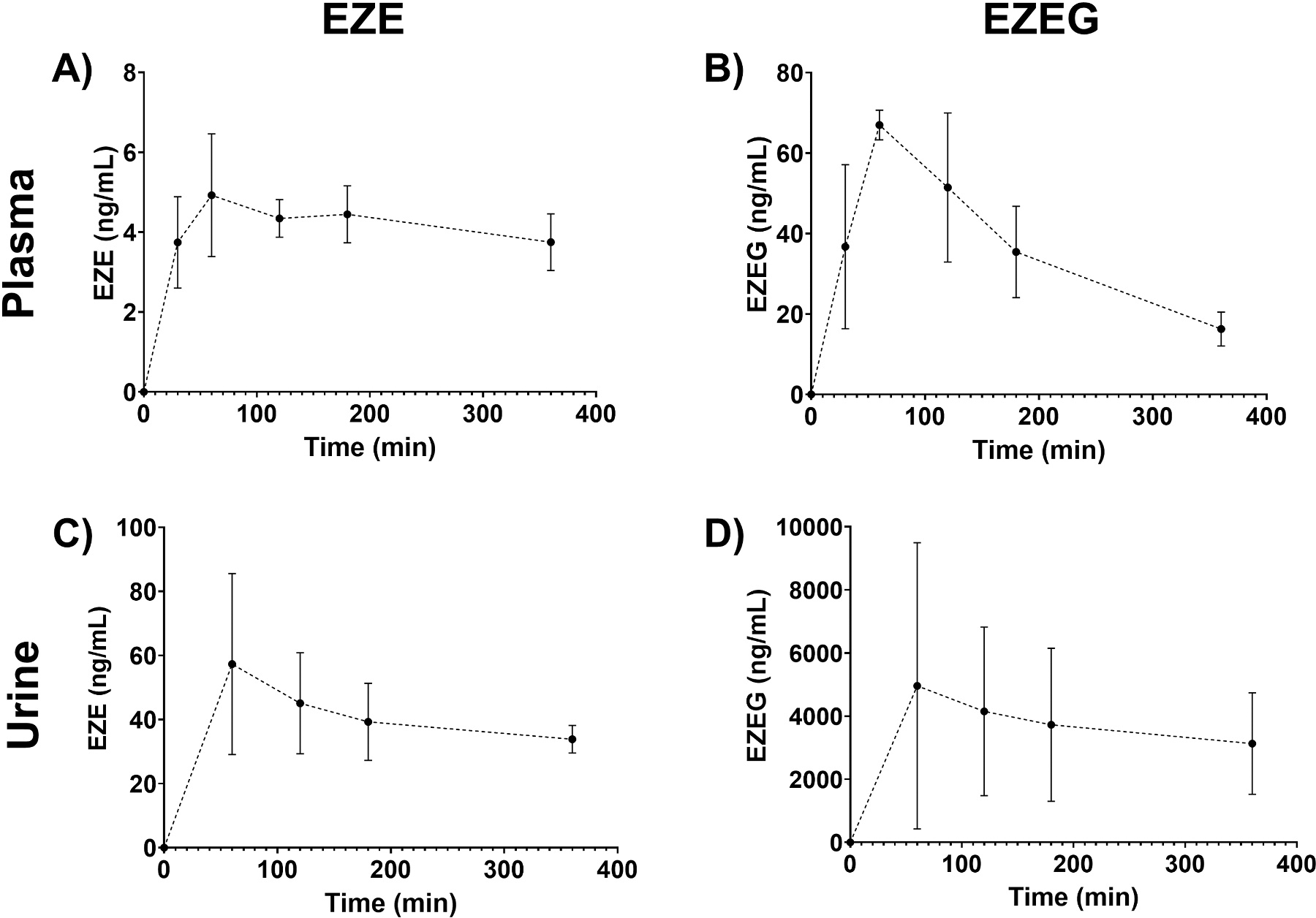
Concentration–time profiles of ezetimibe (EZE) and ezetimibe glucuronide (EZEG) in human volunteers. Following a single oral dose of 1 mg EZE, plasma and urine samples were collected over 6 h and analyzed using the validated LC–MS/MS method. Panels show (A) plasma EZE, (B) plasma EZEG, (C) urine EZE, and (D) urine EZEG, with urine data presented as spot concentrations. Data are shown as mean ± SD (n = 3).

Application of the validated method enabled reliable quantification of both EZE and EZEG in human plasma and urine collected from patients with hepatic impairment of unknown etiology. Plasma concentrations of EZE ranged from 3 to 6 ng/mL (Fig. 2A), while EZEG concentrations ranged from 7 to 73 ng/mL across the sampling period (Fig. 2B). In the urine, EZE concentrations ranged from 31 to 89 ng/mL (Fig. 2C), while EZEG concentrations ranged from 1667 to 10,130 ng/mL (Fig. 2D). These results demonstrate the suitability of the method for quantifying both the parent drug and its glucuronide metabolite over a 6 h sampling period following a single 1 mg oral dose.

## 4. Conclusions

A rapid, sensitive, and specific LC–MS/MS method was developed and validated for the simultaneous quantification of EZE and its glucuronide metabolite, EZEG, in human plasma and urine. Optimization in negative ion mode, using an ACN–water mobile phase containing 0.1% formic acid and a segmented gradient, enabled efficient chromatographic separation and reproducible peak shapes. Protein precipitation with methanol provided effective extraction with consistent recovery and minimal matrix effects across matrices. The method demonstrated adequate specificity, linearity (r^2^ ≥ 0.99), accuracy, and precision, with lower limits of quantification of 1.5 ng/mL in plasma and 3–5 ng/mL in urine. EZE and EZEG were stable under all tested conditions, supporting reliable handling and storage. Overall, this method is suitable for pharmacokinetic, clinical, and translational studies involving EZE and its glucuronide metabolite.

## Supplementary Material

1

2

3

## Figures and Tables

**Table 1:** Mass spectrometer parameters used for detection of EZE and EZEG.

Q1	Q3	RT (min)	Identity	DP (V)	CE (V)

408	271	3.3	Ezetimibe 1	−140	−22
408	139	3.3	Ezetimibe 2	−140	−20
412	275	3.3	Ezetimibe d_4_	−140	−22
584	271	3.1	Ezetimibe-gluc 1	−100	−40
584	139	3.1	Ezetimibe-gluc 2	−100	−38
588	271	3.1	Ezetimibe-gluc d_4_	−100	−40

**Table 2:** Precision and accuracy of EZE and EZEG in human plasma.

		Intra-day run			Inter-day runs		
Analyte	Actual Concentration (ng/mL)	Calculated Concentration (ng/mL) (mean ± SD)	CV (%)	Accuracy (%) (mean ± SD)	Calculated Concentration (ng/mL) (mean ± SD)	CV (%)	Accuracy (%) (mean ± SD)

EZE	1.5	2 ± 0.07	4.17	113.72 ± 4.74	2 ± 0.10	6.17	103.52 ± 6.39
	3	3 ± 0.32	11.16	94.93 ± 10.59	3 ± 0.24	7.76	98.51 ± 8.73
	500	492 ± 42	8.59	98.37 ± 8.45	493 ± 37	7.52	98.62 ± 7.42
	900	940 ± 39	4.10	104.43 ± 4.28	898 ± 72	7.95	99.82 ± 7.94
EZEG	1.5	3 ± 0.22	6.84	95.10 ± 6.50	2 ± 0.12	7.68	101.09 ± 7.77
	3	11 ± 1.28	4.47	92.78 ± 4.15	3 ± 0.17	5.83	99.63 ± 5.81
	500	486 ± 26	9.57	97.26 ± 9.21	492 ± 38	7.75	98.32 ± 7.62
	900	908 ± 39	6.91	100.92 ± 6.97	895 ± 60	6.71	99.43 ± 7.20

**Table 3:** Precision and accuracy of EZE and EZEG in human urine.

		Intra-day run			Inter-day runs		
Analyte	Actual Concentration (ng/mL)	Calculated Concentration (ng/mL) (mean ± SD)	CV (%)	Accuracy (%) (mean ± SD)	Calculated Concentration (ng/mL) (mean ± SD)	CV (%)	Accuracy (%) (mean ± SD)

EZE	5	5 ± 0.73	15.26	95.08 ± 14.51	5 ± 0.27	5.39	100.29 ± 5.41
	15	15 ± 0.36	2.50	96.98 ± 2.42	15 ± 0.53	3.56	99.88 ± 3.07
	500	460 ± 62	13.40	91.94 ± 12.32	488 ± 70	14.27	97.57 ± 13.93
	850	850 ± 22	2.60	99.99 ± 2.60	824 ± 69	8.42	96.96 ± 8.17
EZEG	3	3 ± 0.22	6.64	109.13 ± 7.25	3 ± 0.38	11.97	104.99 ± 12.57
	12	11 ± 1.28	11.55	92.41 ± 10.67	12 ± 0.91	7.72	98.43 ± 7.60
	500	490 ± 62	12.69	97.98 ± 12.43	524 ± 59	11.16	104.89 ± 11.71
	850	930 ± 56	5.97	109.37 ± 6.53	888 ± 75	8.45	104.43 ± 8.82

## Appendix A. Supporting information

Supplementary data associated with this article can be found in the online version at doi:10.1016/j.jpba.2026.117455.
