# Supplementary material for: Detection and quantification of ezetimibe and its major glucuronide in patients with hepatic impairment via liquid chromatography-tandem mass spectrometry
Source: J Pharm Biomed Anal. Author manuscript; Available in PMC 2026 Apr 10. (PMC13067950; doi:10.1016/j.jpba.2026.117455)
Supplement: 1 [file NIHMS2157090-supplement-1.docx]

**Supplemental Table 1.** Stability of EZE and EZEG in human plasma under various storage conditions.

| n=5 | Analyte | Actual Concentration (ng/mL) | Calculated Concentration (ng/mL) | StdDev | Accuracy (%) | StdDev | CV (%) |
| --- | --- | --- | --- | --- | --- | --- | --- |
| Autosampler (4°C) | EZE | 1.5 | 2 | 0.03 | 111.44 | 2.00 | 1.79 |
|  |  | 3 | 3.00 | 0.23 | 99.97 | 7.55 | 7.55 |
|  |  | 500 | 506 | 18 | 101.28 | 3.66 | 3.61 |
|  |  | 900 | 945 | 17 | 105.03 | 1.88 | 1.79 |
|  | EZEG | 1.5 | 2 | 0.15 | 105.00 | 9.89 | 9.42 |
|  |  | 3 | 3 | 0.30 | 94.43 | 9.97 | 10.56 |
|  |  | 500 | 482 | 20 | 96.36 | 3.99 | 4.15 |
|  |  | 900 | 943 | 26 | 104.76 | 2.89 | 2.76 |
| Room Temp (25°C) | EZE | 1.5 | 1 | 0.17 | 98.02 | 11.42 | 11.66 |
|  |  | 3 | 3 | 0.27 | 96.69 | 9.10 | 9.42 |
|  |  | 500 | 493 | 40 | 98.61 | 8.06 | 8.17 |
|  |  | 900 | 863 | 57 | 95.92 | 6.32 | 6.59 |
|  | EZEG | 1.5 | 2 | 0.10 | 102.14 | 6.78 | 6.64 |
|  |  | 3 | 3 | 0.28 | 101.71 | 9.44 | 9.28 |
|  |  | 500 | 471 | 43 | 94.19 | 8.63 | 9.16 |
|  |  | 900 | 812 | 40 | 90.21 | 4.40 | 4.88 |
| Freeze Thaw (3X) | EZE | 1.5 | 2 | 0.11 | 102.01 | 7.30 | 7.16 |
|  |  | 3 | 3 | 0.33 | 100.27 | 11.05 | 11.02 |
|  |  | 500 | 523 | 19 | 104.56 | 3.71 | 3.55 |
|  |  | 900 | 850 | 47 | 94.43 | 5.26 | 5.57 |
|  | EZEG | 1.5 | 2 | 0.09 | 105.26 | 5.84 | 5.55 |
|  |  | 3 | 3 | 0.11 | 105.53 | 3.81 | 3.61 |
|  |  | 500 | 520 | 20 | 104.02 | 4.08 | 3.93 |
|  |  | 900 | 931 | 89 | 103.48 | 9.92 | 9.59 |
| Long Term  (3+ months in -80°C) | EZE | 1.5 | 2 | 0.15 | 108.48 | 9.68 | 8.92 |
|  |  | 3 | 3 | 0.15 | 108.66 | 5.16 | 4.75 |
|  |  | 500 | 502 | 33 | 100.32 | 6.69 | 6.67 |
|  |  | 900 | 950 | 95 | 105.54 | 10.52 | 9.97 |
|  | EZEG | 1.5 | 1 | 0.03 | 97.79 | 1.84 | 1.88 |
|  |  | 3 | 3 | 0.12 | 96.87 | 4.16 | 4.29 |
|  |  | 500 | 465 | 36 | 93.02 | 7.12 | 7.65 |
|  |  | 900 | 896 | 48 | 99.59 | 5.35 | 5.37 |

**Supplemental Table 2.** Stability of EZE and EZEG in human urine under various storage conditions.

| n=5 | Analyte | Actual Concentration (ng/mL) | Calculated Concentration (ng/mL) | StdDev | Accuracy (%) | StdDev | CV (%) |
| --- | --- | --- | --- | --- | --- | --- | --- |
| Autosampler (4°C) | EZE | 5 | 5 | 0.40 | 99.80 | 8.03 | 8.04 |
|  |  | 15 | 14 | 0.99 | 92.62 | 4.52 | 7.26 |
|  |  | 500 | 475 | 67 | 95.09 | 13.35 | 14.04 |
|  |  | 850 | 798 | 103 | 93.85 | 12.10 | 12.89 |
|  | EZEG | 3 | 3 | 0.18 | 105.80 | 6.03 | 5.70 |
|  |  | 12 | 12 | 0.22 | 106.23 | 1.83 | 1.72 |
|  |  | 500 | 520 | 21 | 104.01 | 4.29 | 4.13 |
|  |  | 850 | 924 | 18 | 108.66 | 2.14 | 1.97 |
| Room Temp (25°C) | EZE | 5 | 5 | 0.40 | 107.95 | 8.06 | 7.47 |
|  |  | 15 | 15 | 1.91 | 100.91 | 9.38 | 12.43 |
|  |  | 500 | 500 | 50 | 100.04 | 9.93 | 9.93 |
|  |  | 850 | 824 | 107 | 96.96 | 12.55 | 12.94 |
|  | EZEG | 3 | 3 | 0.30 | 102.79 | 10.07 | 9.80 |
|  |  | 12 | 11 | 0.39 | 89.63 | 3.22 | 3.59 |
|  |  | 500 | 543 | 21 | 108.54 | 4.28 | 3.94 |
|  |  | 850 | 831 | 80 | 97.78 | 9.40 | 9.62 |
| Freeze Thaw (3X) | EZE | 5 | 5 | 0.40 | 99.65 | 8.00 | 8.03 |
|  |  | 15 | 15 | 1.80 | 98.51 | 7.88 | 12.08 |
|  |  | 500 | 476 | 25 | 95.20 | 4.91 | 5.15 |
|  |  | 850 | 855 | 33 | 100.57 | 3.83 | 3.81 |
|  | EZEG | 3 | 3 | 0.32 | 97.17 | 10.63 | 10.94 |
|  |  | 12 | 13 | 0.94 | 105.78 | 7.85 | 7.42 |
|  |  | 500 | 454 | 32 | 90.89 | 6.36 | 7.00 |
|  |  | 850 | 841 | 37 | 98.89 | 4.30 | 4.35 |
| Long Term  (3+ months in -80°C) | EZE | 5 | 5 | 0.52 | 100.40 | 10.33 | 10.29 |
|  |  | 15 | 14 | 0.90 | 95.41 | 5.98 | 6.27 |
|  |  | 500 | 529 | 38 | 105.82 | 7.54 | 7.13 |
|  |  | 850 | 824 | 49 | 96.96 | 5.75 | 5.93 |
|  | EZEG | 3 | 3 | 0.05 | 92.39 | 1.71 | 1.85 |
|  |  | 12 | 12 | 0.89 | 97.72 | 7.45 | 7.62 |
|  |  | 500 | 503 | 32.40 | 100.65 | 6.48 | 6.44 |
|  |  | 850 | 828 | 48.30 | 97.36 | 5.68 | 5.84 |

**
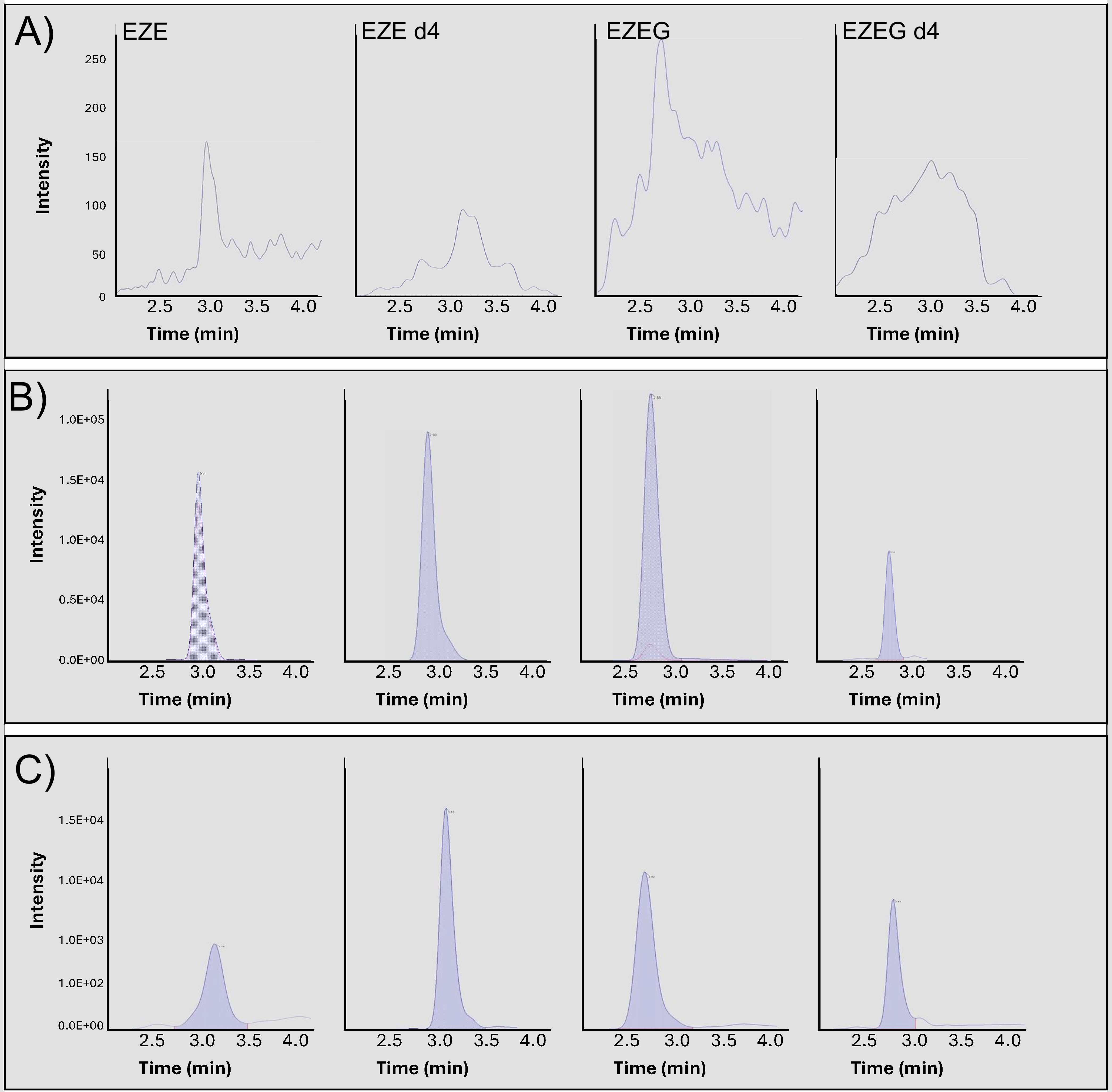
Supplemental Figure 1.** Representative LC-MS/MS chromatograms EZE, EZE d4, EZEG, EZEG d4 in plasma. Representative LC-MS/MS chromatograms for EZE, EZEG, and their respective internal standards (I.S.) in human plasma. (A) blank plasma sample; (B) plasma spiked with 5 ng/mL of EZE and EZEG along with their respective IS; (C) human plasma sample collected from a healthy volunteer following a single oral dose of 1 mg EZE.

**
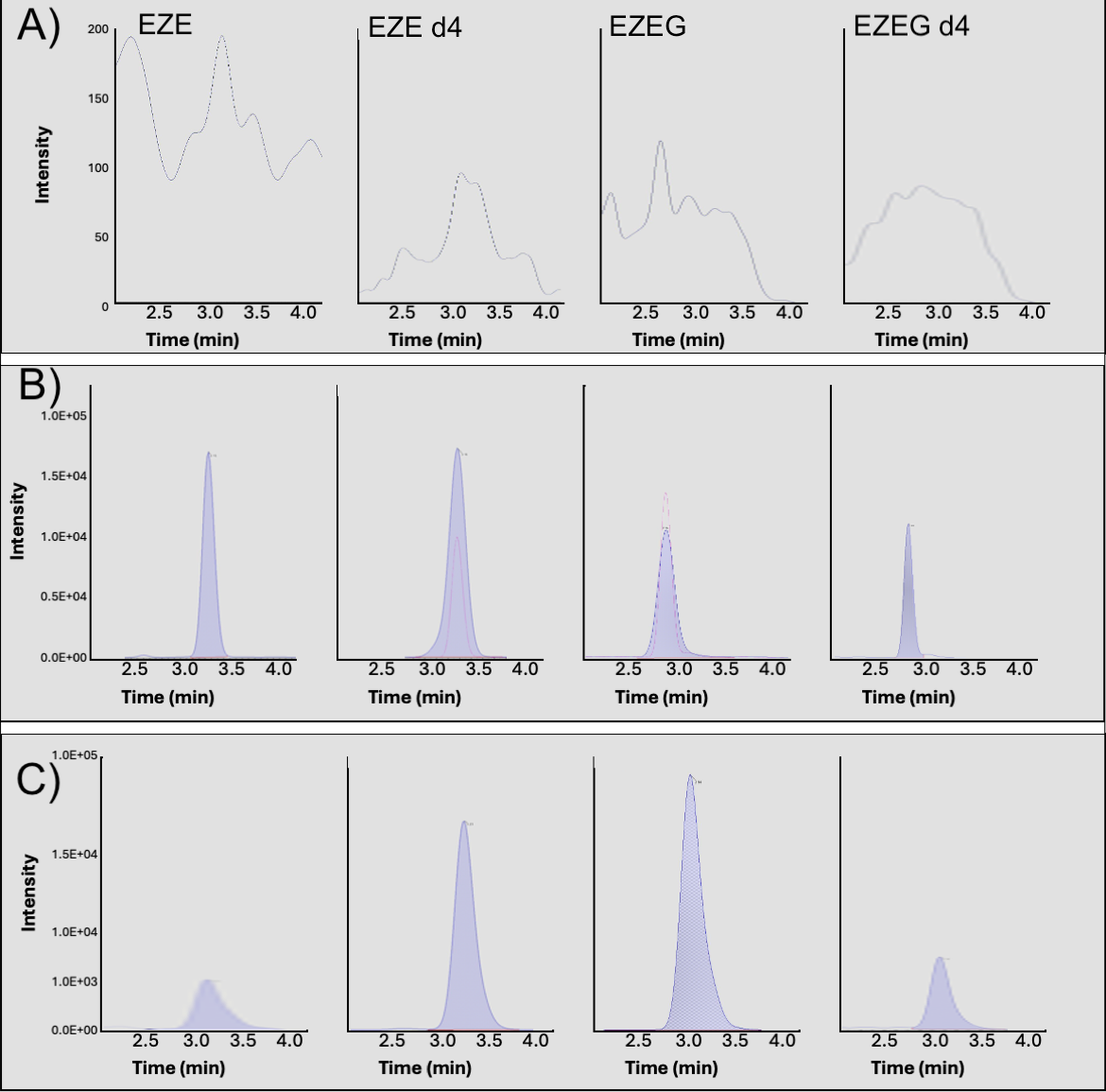
Supplemental Figure 2.** Representative LC-MS/MS chromatograms EZE, EZE d4, EZEG, EZEG d4 in urine. Representative LC-MS/MS chromatograms for EZE, EZEG, and their respective IS in human urine. (A) blank urine sample; (B) urine spiked with 7.5 ng/mL of EZE and EZEG along with IS; (C) urine sample collected from a healthy volunteer following a single oral dose of 1 mg EZE.
