# Supplementary figures and images for: Detection and quantification of ezetimibe and its major glucuronide in patients with hepatic impairment via liquid chromatography-tandem mass spectrometry
Source: J Pharm Biomed Anal. Author manuscript; Available in PMC 2026 Apr 10. (PMC13067950; doi:10.1016/j.jpba.2026.117455)

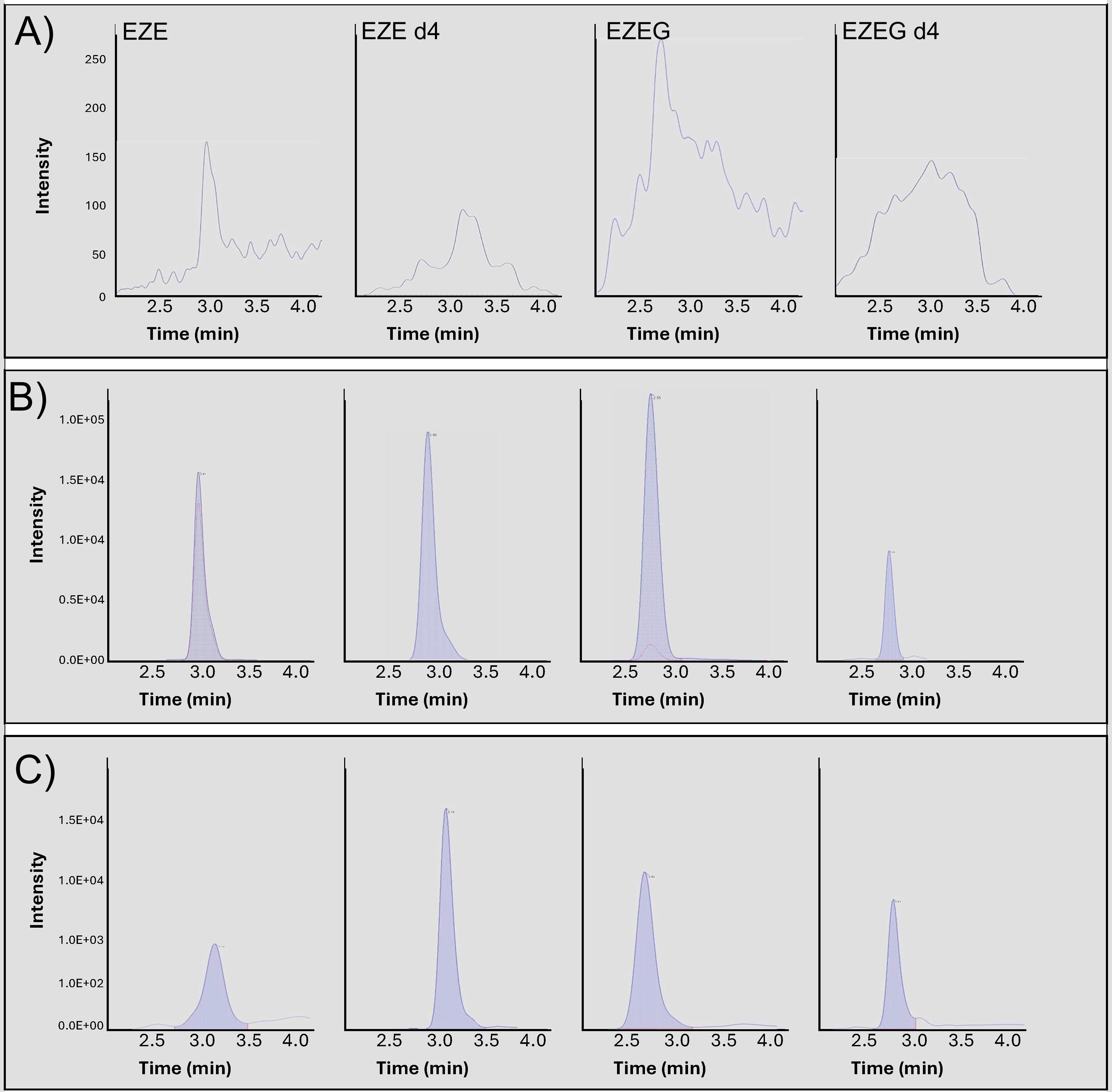

Supplement: 2 [file NIHMS2157090-supplement-2.tiff]

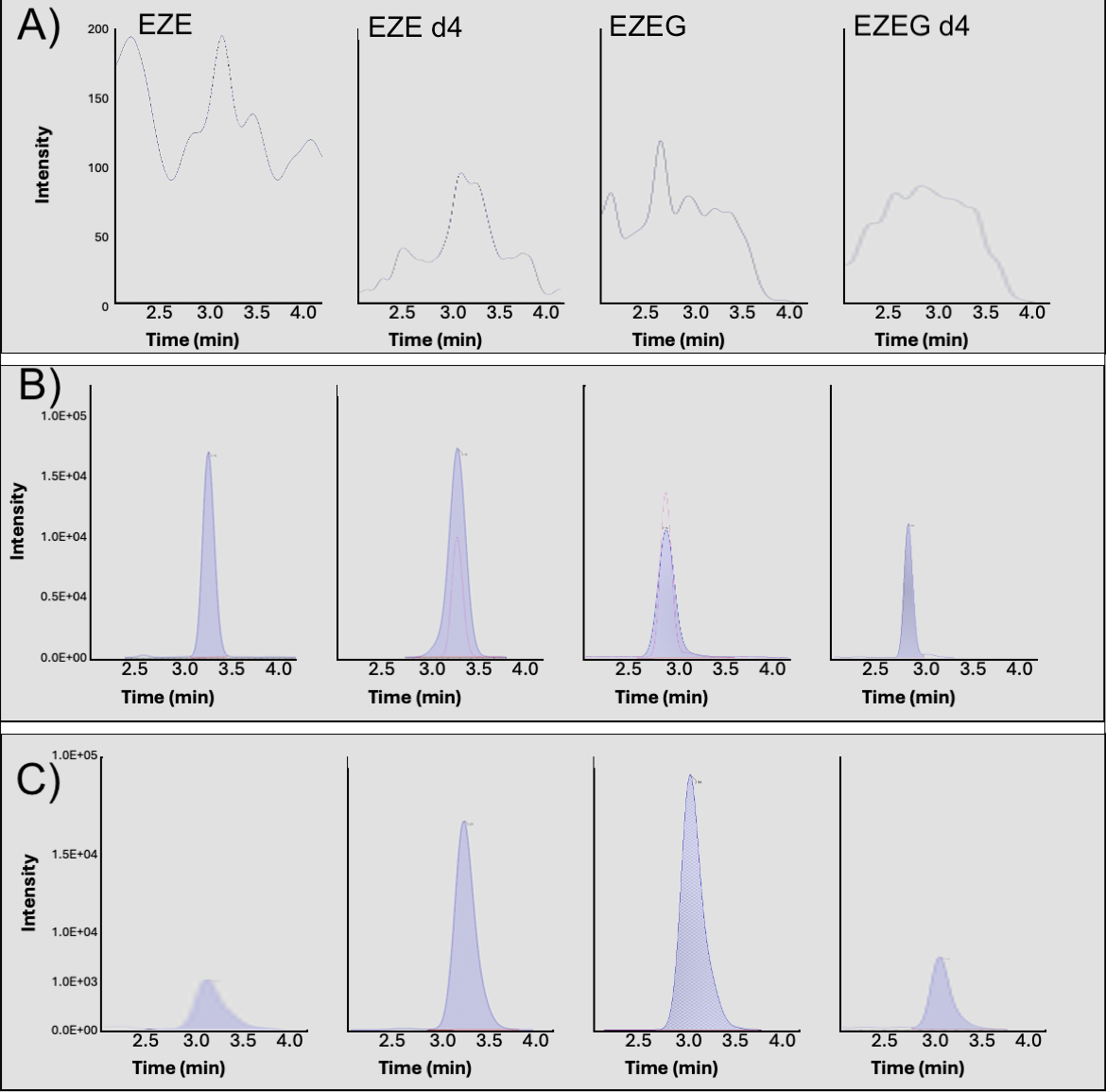

Supplement: 3 [file NIHMS2157090-supplement-3.tiff]
